# The ‘Critical Power’ Concept: Applications to Sports Performance with a Focus on Intermittent High-Intensity Exercise

**DOI:** 10.1007/s40279-017-0688-0

**Published:** 2017-03-22

**Authors:** Andrew M. Jones, Anni Vanhatalo

**Affiliations:** Sport and Health Sciences, College of Life and Environmental Sciences, University of Exeter, Heavitree Road, Exeter, EX12LU UK

## Abstract

The curvilinear relationship between power output and the time for which it can be sustained is a fundamental and well-known feature of high-intensity exercise performance. This relationship ‘levels off’ at a ‘critical power’ (CP) that separates power outputs that can be sustained with stable values of, for example, muscle phosphocreatine, blood lactate, and pulmonary oxygen uptake ($\dot{V}{\text{O}}_{2}$), from power outputs where these variables change continuously with time until their respective minimum and maximum values are reached and exercise intolerance occurs. The amount of work that can be done during exercise above CP (the so-called *W*′) is constant but may be utilized at different rates depending on the proximity of the exercise power output to CP. Traditionally, this two-parameter CP model has been employed to provide insights into physiological responses, fatigue mechanisms, and performance capacity during continuous constant power output exercise in discrete exercise intensity domains. However, many team sports (e.g., basketball, football, hockey, rugby) involve frequent changes in exercise intensity and, even in endurance sports (e.g., cycling, running), intensity may vary considerably with environmental/course conditions and pacing strategy. In recent years, the appeal of the CP concept has been broadened through its application to intermittent high-intensity exercise. With the assumptions that *W*′ is utilized during work intervals above CP and reconstituted during recovery intervals below CP, it can be shown that performance during intermittent exercise is related to four factors: the intensity and duration of the work intervals and the intensity and duration of the recovery intervals. However, while the utilization of *W*′ may be assumed to be linear, studies indicate that the reconstitution of *W*′ may be curvilinear with kinetics that are highly variable between individuals. This has led to the development of a new CP model for intermittent exercise in which the balance of *W*′ remaining ($W_{\text{BAL}}^{\prime }$) may be calculated with greater accuracy. Field trials of athletes performing stochastic exercise indicate that this $W_{\text{BAL}}^{\prime }$ model can accurately predict the time at which *W*′ tends to zero and exhaustion is imminent. The $W_{\text{BAL}}^{\prime }$ model potentially has important applications in the real-time monitoring of athlete fatigue progression in endurance and team sports, which may inform tactics and influence pacing strategy.

## Background to the Power–Time Relationship and the Concept of ‘Critical Power’ (CP)

**Fig. 1:**
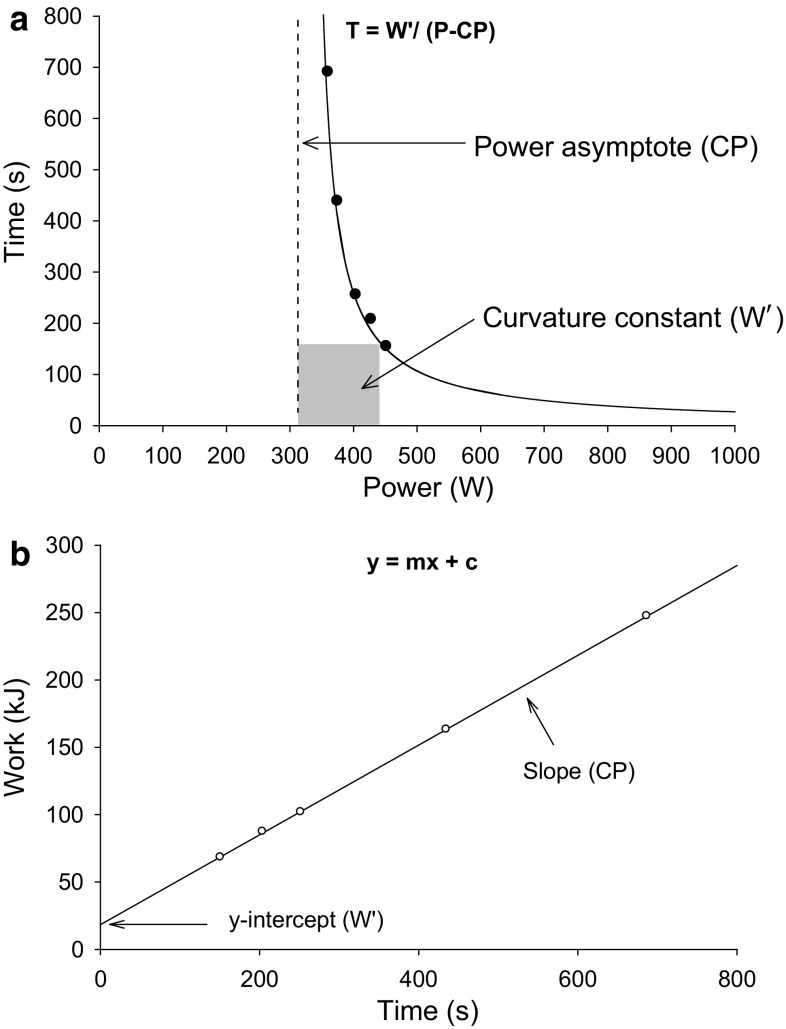
**a** Hyperbolic relationship between power output (*x*-axis) and time (*y*-axis), where the critical power is indicated by the power-asymptote and the *W*′ is the curvature constant; **b** linearized two-parameter critical power model where total work done is plotted against time. In this permutation, the critical power is given by the slope of the regression and the *W*′ is the *y*-intercept. *CP* critical power, *P* power, *T* time, *W*′ curvature constant of power–time relationship

The hyperbolic relationship between power output and the time for which it can be sustained has been well described [1–4]. This relationship is typically established by having a subject complete between three and five separate high-intensity exercise tests on different days, during which they are asked to sustain a fixed external power output for as long as possible. The power outputs are selected to result in ‘exhaustion’ in a minimum of ~2 min and a maximum of ~15 min. The subject’s precise ‘time to the limit of tolerance’ at each of these power outputs is recorded. When power output is subsequently plotted against time, it can be observed that the sustainable power output falls as a function of the exercise duration and that it levels off, or asymptotes, on the abscissa (Fig. 1a). This asymptote has been termed the critical power (CP), which is measured in watts (W), while the curvature of the power–time relationship represents the work capacity available above CP and has been termed *W*′ (measured in kilojoules [kJ]). The information contained in this ‘curvilinear’ power–time relationship can also be expressed if work done in each of the separate exercise bouts is plotted against sustainable time. This results in a more ‘user-friendly’ linear relationship that can be described with the regression equation *y* = *mx* + *c*, where the slope *m* is CP and the intercept *c* is *W*′ (Fig. 1b). It is important to note that while the description given above relates to power output, and the majority of research in this area has employed cycle ergometry, this same hyperbolic relationship exists in other modes of human locomotion, including running [5, 6] and swimming [7]. Here though, the terms critical speed (CS)—or critical velocity (CV), as appropriate—and *D*′, measured in units of m s^−1^ and m, respectively, are used instead of CP and *W*′. Although it is expressed functionally as an external power output, it should be noted that CP reflects a ‘critical metabolic rate’. This point can be illustrated by, for example, manipulating pedal rate during cycling: when pedal rate is elevated and the associated internal work and metabolic cost of cycling is increased, CP is reduced [8].

The power–time relationship is a fundamental concept in exercise physiology for two reasons. First, it provides a framework for exploring and understanding skeletal muscle bioenergetics and the metabolic and cardio-respiratory responses to exercise. Second, it provides a powerful tool for fitness diagnostics, monitoring of the physical impact of interventions such as training and putative ergogenic aids, and performance prediction in continuous high-intensity endurance exercise [9, 10]. Physiologically, CP is important because it defines the boundary between discrete domains of exercise intensity. Below CP, in the ‘heavy’ intensity domain, steady-state values for muscle metabolism (i.e., phosphocreatine concentration [PCr] and pH), blood [lactate], and pulmonary oxygen uptake ($\dot{V}{\text{O}}_{2}$) can be attained [4, 11–13]. However, above CP, in the ‘severe’ intensity domain, these variables do not demonstrate steady-state behavior. Rather, despite the external power output remaining constant, muscle efficiency is lost, as reflected in the development of the $\dot{V}{\text{O}}_{2}$ slow component [14], and this drives $\dot{V}{\text{O}}_{2}$ to its maximum value ($\dot{V}{\text{O}}_{2\hbox{max} }$) at the limit of tolerance. Exercise in the severe domain is also associated with continuous reductions in muscle [PCr] and pH and a progressive accumulation of blood lactate, the minimum or maximum values of which are also attained at the limit of tolerance [4, 11, 12]. It is interesting that these respective minimum and maximum values are similar irrespective of whether the severe-intensity exercise bout is relatively short (2–3 min) or relatively long (12–15 min) [13, 15], suggesting that the limit of tolerance during such exercise (and therefore the magnitude of *W*′) may coincide with the attainment of a certain intra-muscular and/or systemic milieu that the subject cannot, or is not prepared to, exceed.

It has been proposed that the process of fatigue development during severe-intensity exercise may be linked to the inter-relationships between the recruitment of type II muscle fibers; the reduction of muscle efficiency; changes in muscle substrates and metabolites, which might simultaneously impair muscle contractile function and stimulate mitochondrial respiration; and the development of the $\dot{V}{\text{O}}_{2}$ slow component leading ultimately to the attainment of $\dot{V}{\text{O}}_{{2{ \hbox{max} }}}$ [9, 16]. Collectively, these factors would be expected to dictate the size of *W*′ and the tolerable duration of exercise above CP. These same relationships appear to hold during a 3-min all-out test (3AOT), during which power falls to attain a stable value after ~2 min and $\dot{V}{\text{O}}_{2\hbox{max} }$ is attained [17]. The 3AOT, which was designed to expedite the derivation of the power–time parameters using a single maximal test rather than multiple repeated maximal tests, has been shown to provide valid and reliable estimates of CP (based on the end-test power) and *W*′ (based on the total work done above the end-test power) during cycle exercise [18–20] and, more recently, other exercise modalities [21–23].

The CP may be functionally defined as the highest power output that can be sustained without progressively drawing on *W*′, where the latter represents, at the onset of exercise, a fixed amount of work that can be done when CP is exceeded. If the power output being sustained was considerably above CP such that the tolerable duration of exercise was short, *W*′ would be utilized (or, perhaps more appropriately, accumulated) linearly and at a more rapid rate than would be the case if the power output being sustained was only just above CP and exercise duration was correspondingly longer. While it is tempting to consider *W*′ as an ‘anaerobic’ capacity, comprising energy that may be derived from substrate-level phosphorylation as well as stored O_2_, observations of inter-relationships between CP and *W*′ [19, 20, 24] suggest that this may be an oversimplification.

It is important to appreciate that performance in the severe-intensity domain (which, incidentally, encompasses a rather large swathe of athletic events, for example, in track and field, from 800 m up to perhaps 10,000 m), depends upon both CP and *W*′. While CP will dictate the highest sustainable oxidative metabolic rate, the size of *W*′ will determine the sustainable duration of exercise above that metabolic rate. Knowledge of an athlete’s CP and *W*′ permits a coach or sports scientist to calculate that athlete’s best possible time for a given distance and to consider tactical, positional, and pacing approaches that might optimize performance relative to the athlete’s competitors [9, 10, 25, 26].

While the parameters that may be extracted from the power–time relationship have many valuable applications in sport, a key limitation is that they are conventionally derived entirely on the basis of performance during constant power output exercise. Such a scenario is rather rare in ‘real-world’ sport. Many sports, especially team sports, involve intermittent bouts of high-intensity exercise separated by variable durations of lower-intensity exercise or rest, and even ‘continuous’ sports events often involve variations in pacing due to terrain, environmental conditions, and the tactics employed by the athlete and his or her competitors. Moreover, if the CP concept is to be extended to the prescription and evaluation of training, then it would be advantageous if this could encompass intermittent as well as continuous exercise because most athletic training programs involve both interval training and steady-state aerobic exercise. The purpose of this article is to provide an overview of novel applications of the CP concept in sport with particular emphasis on variable power and intermittent exercise.

## The CP Concept and Variable-Pace Exercise

The CP occurs at a higher absolute and relative intensity than the lactate threshold (LT), which represents the boundary between the ‘moderate’ and heavy-intensity exercise [4, 27]. It may be considered to be more akin to the so-called maximal lactate steady state [28, 29] and may occur at 70–90% $\dot{V}{\text{O}}_{{2{ \hbox{max} }}}$, depending on training status, making it relevant to a wider range of athletic events than the LT. While the LT might still be considered relevant to longer-duration endurance exercise such as the marathon, it should be noted that, in elite endurance athletes, the LT and CP both occur at a relatively high fraction of $\dot{V}{\text{O}}_{{2{ \hbox{max} }}}$ such that these metabolic thresholds are positioned closer together than would be the case in sub-elite athletes [30].

**Fig. 2:**
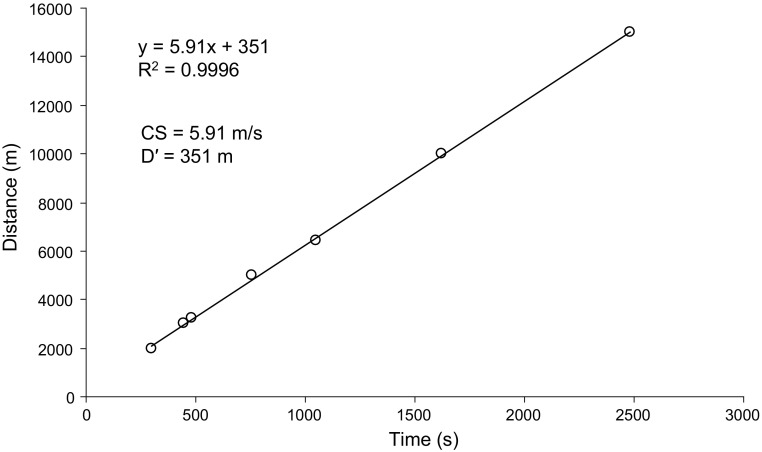
An example of the estimation of critical speed and *D*′ in Haile Gebrselassie, using the linear distance–time model. The distances modelled ranged from 2 km (4:56.1) to 15 km (41:38). Critical speed was 5.91 m s^−1^ and *D*′ was 351 m. *CS* critical speed, *D′* curvature constant of speed–time relationship, *R* ^*2*^ coefficient of determination

We undertook an analysis of marathon performance in relation to estimated CS in elite-level marathon athletes. We retrieved relevant performance information online (International Association of Athletics Federations [IAAF] http://www.iaaf.org/athletes) from 12 elite male marathoners (personal best times ranging from 2:03:38 to 2:08:21). For these 12 runners, we were able to find personal best times over shorter race distances between 1500 m and 15 km; at least four race distances for each athlete were included in the estimation of CS and D′. An example of the estimation of CS and D′ in one of the athletes included in the analysis, Haile Gebrselassie, is provided in Fig. 2. When distance (between 2 and 15 km) is plotted against time taken to complete the distance, it can be seen that the relationship is highly linear (*R* ^2^ > 0.999), with CS being estimated as 5.91 m s^−1^ and *D*′ estimated as 351 m. The individual and group mean values for CS, *D*′, marathon time, and marathon speed are shown in Table 1. The key result of this analysis is that, on average, the elite athletes completed the marathon distance at 96 ± 2% of their CS. This consistency is remarkable for a number of reasons. Different race distances were necessarily used for each athlete, with some athletes not having official personal best times for distances below 10 km; the ‘bias’ towards longer distances in the analysis stretches the applicable range of the speed–time relationship and would tend to lead to an underestimation of CS. Furthermore, personal best times were run at different times in the athletes’ careers, often several years prior to their marathon best performances, and this would be expected to introduce some error into the CS and *D*′ estimates. Finally, the CS estimate was determined using personal best performances over shorter race distances while in a ‘fresh’ state. Long-duration, fatiguing exercise might be expected to result in a reduction in CS, perhaps related to a reduction of running economy [31], such that mean marathon race speed might be even closer to the CS than the analysis suggests. Overall, this comparison of CS from personal best times over shorter distances to best marathon performance indicates that elite marathon athletes may regulate their race pace so as to be in close proximity to the CS. From a bioenergetic perspective, avoiding frequent or protracted excursions beyond CS, except perhaps when tactically necessary or in a sprint finish, would seem sensible to prevent utilization of *D*′ and the associated muscle metabolic and systemic perturbations that would expedite fatigue [10, 11].

**Table 1:** Individual and group mean values for curvature constant of speed–time relationship, critical speed, marathon time, and marathon speed for a selection of elite male marathon runners. The distances modelled ranged from 1500 m to 15 km (IAAF http://www.iaaf.org/athletes)

Athlete	Critical speed (m/s)	Curvature constant of speed–time relationship (m)	Marathon time (h min s)	Mean speed (m/s)	% of critical speed
Patrick Makau Musyoki	5.72	287	2.03:38	5.69	99
Haile Gebrselassie	5.91	351	2.03:59	5.67	96
Eliud Kipchoge	6.04	250	2.04:05	5.67	94
Geoffrey Mutai	5.83	290	2.04:15	5.66	97
Ayele Abshero	5.82	352	2.04:23	5.65	97
Samuel Kamau Wanjiru	5.99	224	2.05:10	5.62	94
Evans Rutto Limo	5.59	616	2.05:50	5.59	100
Khalid Khannouchi	5.70	372	2.05:38	5.60	98
Felix Limo	5.92	298	2.06:14	5.57	94
António Pinto	6.00	231	2.06:36	5.55	93
Steve Jones	5.80	294	2.07:13	5.53	95
Mohamed Farah	5.75	373	2.08:21	5.48	95
Mean	5.84	328	2.05:27	5.61	96
SD	0.14	104	0.01:28	0.07	2

*SD* standard deviation

As described in Sect. 1, the power–time parameters are typically derived from several exhaustive exercise bouts completed at discrete but constant power outputs, with *W*′ so derived being considered as a fixed amount of work that can be done above CP. However, whether *W*′ is indeed fixed when severe-intensity exercise is performed not at a constant power output but with different pacing strategies (e.g., incremental, decremental, all-out, variable) has received limited attention. Chidnok et al. [32] estimated CP and *W*′ from the 3AOT and then calculated the work done above CP during different forms of maximal exercise, including a ramp incremental exercise test, a constant power output test where the predicted exercise duration was 3 min, and a test where the subjects were instructed to complete as much work as possible in 3 min and could choose their own pacing strategy to achieve this goal. The total work done above CP did not significantly differ between conditions, being 16.5 ± 4.0 kJ for 3AOT, 16.4 ± 4.0 kJ for incremental exercise, 16.6 ± 7.4 kJ for constant power output exercise, and 15.3 ± 5.6 kJ for self-paced exercise. The $\dot{V}{\text{O}}_{{2{ \hbox{max} }}}$ value that was attained also did not differ between the four conditions. The authors concluded that the limit of tolerance during severe-intensity exercise coincides with the achievement of the same $\dot{V}{\text{O}}_{{2{ \hbox{max} }}}$ and completion of the same amount of work above CP, irrespective of the work rate forcing function or pacing strategy (imposed or self-selected). This suggests that the physiological underpinnings of severe-intensity exercise performance are highly predictable (based on the power–time relationship parameters) and are not affected by pacing strategy.

However, an important caveat to this interpretation is that the work done above CP calculated by Chidnok et al. [32] assumed that CP itself was not changed in the different exercise tests. This assumption was recently tested by Black et al. [33], who established CP and *W*′ both from a conventional series of constant power output (CPO) tests and from a series of time trials (TTs) during which subjects could choose their pacing strategy to complete the set distances as quickly as possible. Consistent with Chidnok et al. [32], *W*′ did not differ between protocols (TT 18.1 ± 5.7 vs. CPO 20.6 ± 7.4 kJ); however, CP was significantly greater when the prediction trials were self-paced (TT 265 ± 44 W vs. CPO 250 ± 47 W). A higher CP alongside a similar *W*′ would translate into improved severe-intensity exercise performance. These results suggest that field-based TT performance times may not always be accurately estimated from conventional laboratory-based constant power output protocols.

It has been reported that a ‘fast-start’ pacing strategy may accelerate the rise in $\dot{V}{\text{O}}_{2}$ following the onset of exercise relative to a more even pacing strategy [34–36]. It has also been reported that the parameters of the power–time relationship are related to other markers of cardio-respiratory fitness [37]. Of particular note is that, across subjects, CP is related to fast $\dot{V}{\text{O}}_{2}$ on-kinetics [38] and *W*′ is related to the magnitude of the $\dot{V}{\text{O}}_{2}$ slow component [17, 38]. In the study by Black et al. [33], *V*O_2_ on-kinetics were faster during TT than during CPO trials, and this improvement was correlated with the higher CP measured during TT compared with CPO trials. Although *W*′ did not significantly differ between conditions, the change in CP was inversely correlated with the change in *W*′. These results suggest that CP and *W*′ might not be entirely independent entities but rather that they comprise features of an integrated bioenergetic system [9, 13, 16, 17, 20]. Faster $\dot{V}{\text{O}}_{2}$ on-kinetics as a consequence of a given intervention, such as a fast-start pacing strategy, might reduce the initial O_2_ deficit, elevate CP, and reduce the $\dot{V}{\text{O}}_{2}$ slow component and *W*′. Consistent with this, an endurance training intervention might be expected to result in faster $\dot{V}{\text{O}}_{2}$ on-kinetics and a reduced $\dot{V}{\text{O}}_{2}$ slow component amplitude post-training compared with pre-training for the same severe-intensity power output [39–41]. In this context, it is interesting to note that endurance training consistently increases CP but also tends to reduce *W*′ [20, 24]. This latter finding would not be anticipated if *W*′ is a fixed ‘anaerobic’ work capacity. Reciprocal changes in CP and *W*′ as a consequence of an experimental intervention are consistent with the view that *W*′ represents the amount of work that can be done above CP prior to the attainment of $\dot{V}{\text{O}}_{{2{ \hbox{max} }}}$ and exercise intolerance [16, 17]. In this regard, the utilization of *W*′ would be closely associated with the development of the $\dot{V}{\text{O}}_{2}$ slow component (and its associated muscle metabolic stimuli) during severe-intensity exercise. This may explain why interventions such as endurance training [20, 24] and hyperoxic or hypoxic gas inspiration [13, 42], which alter the ‘metabolic range’ between CP and $\dot{V}{\text{O}}_{2\hbox{max} }$, also result in changes to *W*′.

## Physiological Responses in Recovery from Severe-Intensity Exercise

The CP concept predicts that recovery from exhaustive severe-intensity exercise requires power output to be reduced below CP. This is because the finite *W*′ is only utilized above CP; because CP reflects the highest sustainable oxidative metabolic rate, exercise below CP should theoretically permit some ‘oxidative metabolic reserve’ to be used for recovery processes (e.g., replenishment of high-energy phosphates, H^+^ clearance). Coats et al. [43] investigated this by asking six subjects to cycle at a power output leading to intolerance in 360 s and then having them continue for as long as possible (up to a maximum of 20 min) when the power output was reduced to 110% CP (i.e., still within the severe-intensity domain), 90% CP (heavy-intensity exercise), or 80% LT (moderate-intensity exercise). The results were partially consistent with the hypothesis: when power output was reduced to 80% LT, all subjects were able to complete a further 20 min of exercise; when power was reduced to 90% CP, two subjects completed 20 min of exercise whereas the other four could only tolerate a further ~10 min; and when power was reduced to 110% CP, the subjects could only tolerate a further ~30 s of exercise. While the results support the notion that recovering *W*′ after exhaustive severe-intensity exercise necessitates a sub-CP power output, the inter-subject variability in exercise tolerance at 110% CP and 90% CP suggests that error in the estimation of CP and/or changes in CP as part of the fatigue process might also have impacted the results.

**Fig. 3:**
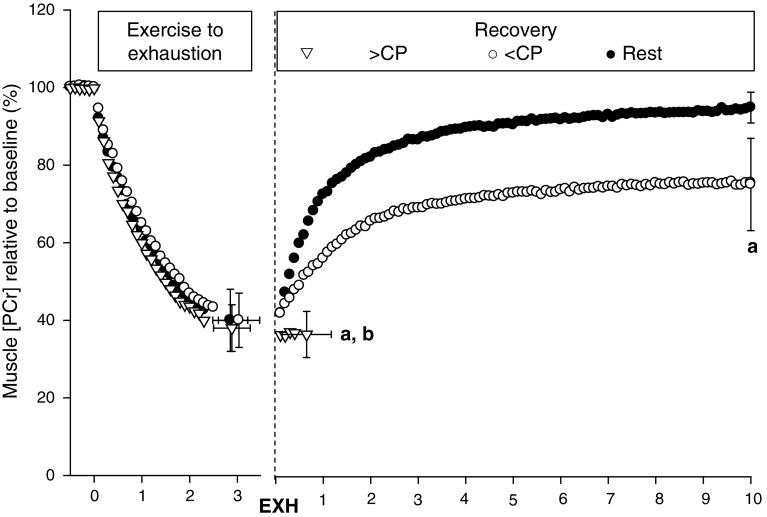
Muscle phosphocreatine responses to constant power output severe-intensity exercise immediately followed by passive recovery (*black circles*), exercise <CP (*white circles*) or exercise >CP (*white triangles*). **a** End-recovery muscle [PCr] was lower in >CP and <CP recovery conditions compared with rest (*p* < 0.05). **b** End-recovery muscle [PCr] was significantly lower for >CP condition compared with rest and <CP recovery (*p* < 0.05). Figure has been re-drawn based on data from Chidnok et al. [44]. *CP* critical power, *EXH* exhaustion, [Pcr] phosphocreatine concentration

Chidnok et al. [44] tested the hypothesis that muscle high-energy phosphate compounds and metabolites related to the fatigue process would be recovered during exercise performed below but not above CP and that these changes would influence the capacity to continue exercise. In this study, subjects completed knee-extension exercise to exhaustion (for ~180 s) on three occasions, followed by a reduction of power output to severe-intensity exercise, heavy-intensity exercise, or a 10-min passive recovery period, while the muscle metabolic responses to exercise were assessed using ^31^P magnetic resonance spectroscopy (^31^P-MRS; Fig. 3). There was a significant difference between the sustainable exercise duration during the recovery from exhaustive severe-intensity exercise between the <CP and >CP conditions (at least 10 min and ~39 s, respectively). During passive recovery and <CP recovery exercise, muscle [PCr] increased rapidly, reaching ∼96 and ∼76% of baseline values, respectively, after 10 min. At these recovery intensities, muscle pH also increased rapidly. However, during >CP exercise, neither muscle [PCr] nor pH recovered, remaining at the nadir reached at the termination of the initial exercise bout. These results confirm that the muscle metabolic dynamics in recovery from exhaustive severe-intensity exercise differ according to whether subsequent exercise is performed below or above CP and are consistent with CP representing an important intramuscular metabolic threshold that dictates the accumulation of fatigue-related metabolites and the capacity to tolerate high-intensity exercise. However, it is interesting that exercise could be continued (albeit for a relatively brief period) when power output was reduced but remained above CP following exercise intolerance. This result, which may be considered surprising but is consistent with those of Coats et al. [43], might indicate that severe-intensity exercise tolerance is related not just to the size of *W*′ but also to the rate at which *W*′ is utilized. Changing the rate of *W*′ utilization by reducing the power output would be expected to reduce the rate of muscle substrate depletion and metabolite accumulation, and it is intriguing that this enables exercise to be continued, albeit briefly. In this regard, it is interesting that decremental exercise performed above CP (i.e., wherein an initially high power output is gradually reduced with time) results in greater exercise tolerance than either incremental or constant power output exercise [36]. Other evidence that *W*′ is more than simply a capacity is provided by the observation that when *W*′ is reduced by prior severe-intensity exercise, the maximal rate of *W*′ utilization (as implied by the peak power output attained in the 3AOT) is correspondingly reduced [45]. The introduction of a third parameter, representing maximal instantaneous power, into the model may enable a better description/prediction of physiological behavior in these situations [46]. This three-parameter model implies that the maximal power at any time is proportional to the *W*′ remaining and that exercise intolerance may not necessarily coincide with complete expenditure of the *W*′ [46].

## Application of the CP Concept to Intermittent Exercise

Understanding the rate and magnitude of recovery processes following the termination of severe-intensity exercise is an important step towards the application of the CP concept in intermittent exercise during which intense bouts of exercise are interspersed with periods of rest or lower-intensity exercise. This may be useful in enabling a better appreciation of the physiological factors that limit intermittent exercise performance and therefore also in performance prediction and training prescription in this form of exercise.

Morton and Billat [47] were the first to develop the CP model for intermittent exercise. These authors recognized that intermittent exercise tolerance is a function of four independent variables: work interval power output (*P*_W_), work interval duration (*D*_W_), recovery interval power output (*P*_R_), and recovery interval duration (*D*_R_). For the model to be valid, the power output for the work intervals must be above CP, the power output for the recovery intervals must be below CP, and the mean power output for the session must be above CP (if not, the exercise could theoretically continue indefinitely). Within these restrictions, Morton and Billat [47] showed that if one of *P*_W_, *D*_W_, or *P*_R_ is increased while the other variables are held constant, exercise tolerance is reduced; however, if *D*_R_ is increased while the other variables are held constant, exercise tolerance is increased. It is assumed that (1) a greater *P*_W_ and/or *D*_W_ will lead to a greater rate and/or total utilization of *W*′, (2) a smaller *P*_R_ and/or a greater *D*_R_ will lead to a greater rate and/or total reconstitution of *W*′, and (3) the utilization and reconstitution of *W*′ proceed in a linear fashion. Exercise tolerance during intermittent exercise (*t*) is therefore given by the following equation, where n is the total number of complete work + rest cycles:

1 $$ t = n\left( {D_{\text{W}} + D_{\text{R}} } \right) + [W{^{\prime}} - n[\left( {P_{\text{W}} {-}{\text{CP}}} \right)D_{\text{W}} {-}\left( {{\text{CP}}{-}P_{\text{R}} } \right)D_{\text{R}} ]/\left( {P_{\text{W}} - {\text{CP}}} \right) $$

Morton and Billat [47] also showed that values for CP and *W*′ parameters were different (lower and higher, respectively) when derived from intermittent exercise than from continuous exercise. This indicates that CP and *W*′ measured during conventional continuous constant power output exercise bouts might not necessarily provide relevant information on performance during intermittent exercise.

**Fig. 4:**
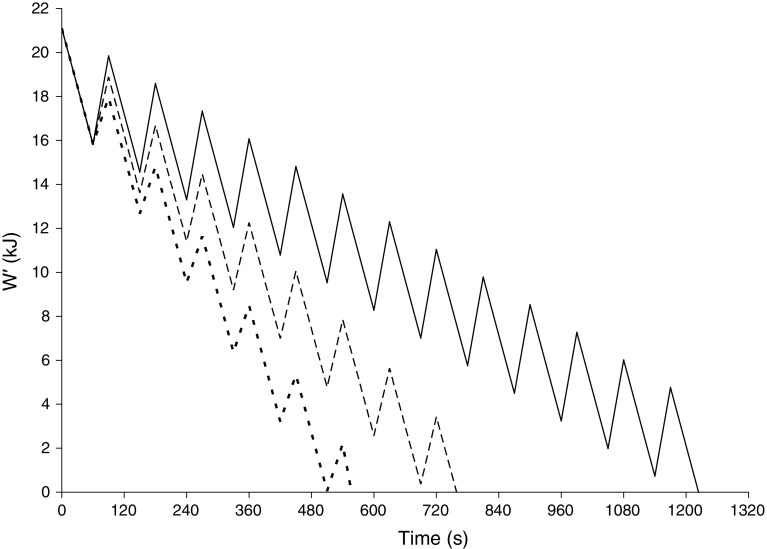
Relationships between *W*′ depletion and reconstitution with different recovery intensities during intermittent exercise: severe–heavy (*dotted line*), severe–moderate (*dashed line*), and severe–light (*solid line*). Figure has been re-drawn based on data from Chidnok et al. [48]. *W*′ curvature constant of power–time relationship

To test some of the assumptions inherent in the model of Morton and Billat [47], Chidnok et al. [48] determined CP and *W*′ with the 3AOT and then asked subjects to complete a severe-intensity constant power output cycle test (S-CPO) and four further tests to exhaustion using different intermittent protocols to the limit of tolerance: severe–severe (S–S), severe–heavy (S–H), severe–moderate (S–M), and severe–light (S–L). In this set of experiments, *P*_W_ was held constant, *D*_W_ was held constant at 60 s, and *D*_R_ was held constant at 30 s; only *P*_R_ was manipulated. The tolerable duration of exercise in S-CPO was ~384 s and, as hypothesized, exercise tolerance was progressively increased when *P*_R_ was reduced (i.e., by 47, 100 and 219%, for S–H, S–M, and S–L, respectively). The greater exercise tolerance at lower *P*_R_ was linearly related to the total work done above CP, which, compared with S-CPO (~23 kJ), was significantly and progressively greater for S–H, S–M, and S–L. However, consistent with expectations, the total work done above CP was similar for S–S and S-CPO and did not differ from *W*′ measured in the 3AOT. Using the known values of CP, *P*_W_, and *D*_W_, Chidnok et al. [48] calculated how much *W*′ was utilized in each work interval and, knowing the tolerable exercise duration for each protocol and assuming that *W*′ was fully utilized at the limit of tolerance, also calculated the extent of *W*′ reconstitution during each recovery interval (Fig. 4). Because *D*_R_ did not differ between the intermittent exercise protocols, the results indicate that *W*′ is reconstituted more rapidly when there is a greater difference between CP and *P*_R_.

**Fig. 5:**
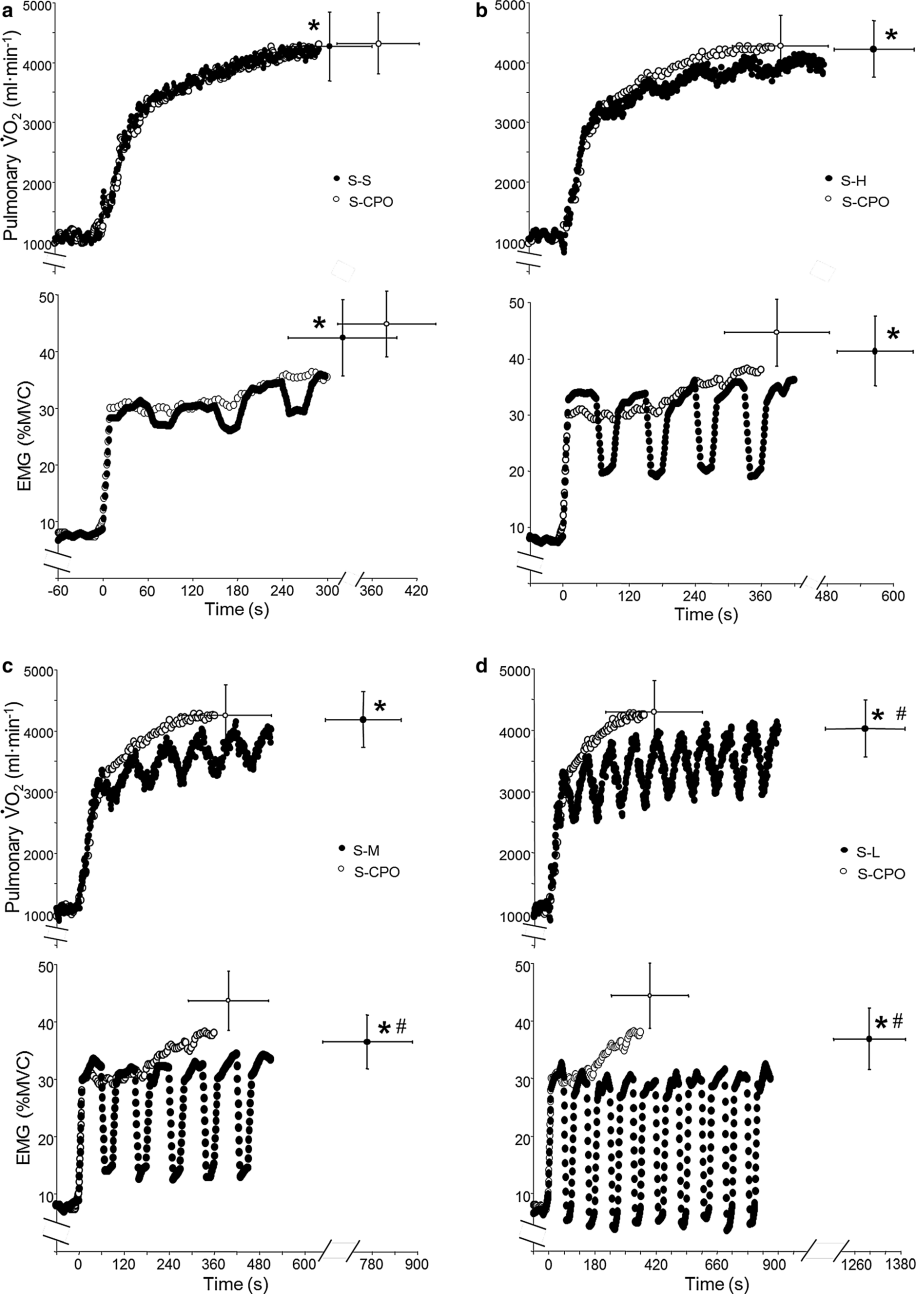
$\dot{V}{\text{O}}_{2}$ and EMG responses during severe constant power output (S-CPO) exercise and intermittent severe-intensity exercise with recovery power output (S-CWR) at severe (**a**), heavy (**b**), moderate (**c**), or light intensity (**d**). Open symbols represent responses during constant power output severe-intensity exercise and closed symbols represent responses when severe-intensity exercise is interspersed with recovery intervals in the severe (S–S), heavy (S–H), or moderate (S–M) domains and for light (20 W) exercise (S–L). *Time to exhaustion differed significantly from S-CWR (*p* < 0.05). ^#^ End-exercise $\dot{V}{\text{O}}_{2}$ and EMG was significantly lower than S–M and S–L (*p* < 0.05). Figure has been re-drawn based on data from Chidnok et al. [48]. *EMG* electromyogram, *MVC* maximal voluntary contraction, $\dot{V}{\text{O}}_{2}$ oxygen uptake

The results of Chidnok et al. [48] indicate that, when recovery intervals in intermittent exercise are performed below CP, exercise tolerance is improved in proportion to the reconstitution of the finite *W*′. Interestingly, compared with S-CPO, the slope of the relationship between both $\dot{V}{\text{O}}_{2}$ and time and the integrated electromyogram (iEMG) and time were systematically reduced for S–H, S–M, and S–L (Fig. 5). Thus, the physiological bases to exercise intolerance during intermittent exercise might be similar to those believed to be operant during continuous exercise and relate to the inter-relationships between substrate depletion and metabolite accumulation and their role in the development of fatigue, recruitment of additional (type II) muscle fibers, and a loss of muscle efficiency manifest in the $\dot{V}{\text{O}}_{2}$ slow component, which results in the attainment of $\dot{V}{\text{O}}_{{2{ \hbox{max} }}}$ at the limit of tolerance. Chidnok et al. [48] found that the enhanced exercise tolerance with lower *P*_R_ was associated with a blunted increase in both $\dot{V}{\text{O}}_{2}$ and iEMG with time, indicating a relationship between the overall rate of *W*′ utilization and the accumulation of fatigue. The trajectory of $\dot{V}{\text{O}}_{2}$ towards $\dot{V}{\text{O}}_{2\hbox{max} }$ appears to be an important portent of fatigue development during both continuous and intermittent exercise when the mean power output exceeds CP [16, 48, 49].

Consistent with Morton and Billat [47], Chidnok et al. [48] found that, compared with the values derived from the 3AOT, CP was significantly reduced and *W*′ was significantly increased when derived from intermittent exercise protocols. While the higher *W*′ is to be expected given the opportunity afforded by recovery intervals for the reconstitution of *W*′ during intermittent exercise, the lower CP is more difficult to explain and requires further investigation. It is possible that the lower CP is a direct consequence of the more substantial non-oxidative contribution to energy turnover during intermittent exercise due to the relatively short duration of the work bouts and/or to a greater energy cost of repeated stop–start activity. This reciprocity between CP and *W*′ has also been reported following endurance training interventions [20, 24], with the breathing of hypoxic and hyperoxic gas mixtures [13, 42], and during blood flow occlusion [50]. Collectively, these observations suggest that CP and *W*′ should not be considered as separate ‘aerobic’ and ‘anaerobic’ entities but rather as components of an integrated bioenergetic system [9, 13, 16, 17, 20].

Chidnok et al. [51] extended their observations on the effects of altering *P*_R_ on *W*′ and exercise tolerance during intermittent cycling by investigating the influence of altering D_R_ on muscle metabolic responses (measured with ^31^P-MRS) and exercise tolerance during knee extension exercise. In this study, *P*_W_ and *D*_W_ were held constant, but subjects were asked to exercise to the limit of tolerance on three occasions with passive (i.e., *P*_R_ = 0 W) recovery durations of 18, 30, or 48 s. The tolerable duration of exercise was ~304, ~516, and ~847 s for the 18-, 30-, and 48-s recovery protocols, respectively. The restoration of muscle [PCr] (as well as adenosine diphosphate [ADP] and inorganic phosphate [*P*_i_]) during recovery was greatest, intermediate, and least for 48, 30, and 18 s of recovery, respectively. The degree of muscle [PCr] restoration was approximately twice as large when 48 versus 18 s of passive recovery was allowed. Consistent with Chidnok et al. [48], the total work done above CP was significantly greater for all intermittent protocols compared with the subjects’ *W*′. This difference became progressively greater as *D*_R_ was increased and was significantly correlated with the mean magnitude of muscle [PCr] reconstitution between work intervals. These results indicate that during intermittent high-intensity exercise, recovery intervals allow the concentrations of high-energy phosphates to be partially restored, with the degree of restoration being related to the duration of the recovery interval. Consequently, the ability to perform work above CP during intermittent high-intensity exercise and, therefore, exercise tolerance, increases when recovery-interval duration is extended.

Despite almost threefold differences in time to the limit of tolerance (~5, 10, and 15 min for 18, 30, and 48 s of recovery, respectively) in the study of Chidnok et al. [51], the muscle metabolic environment (i.e., [PCr], [ADP], [*P*_i_], pH) at the limit of tolerance did not differ significantly between the three intermittent exercise protocols. The metabolic milieu at the point of exercise intolerance during severe-intensity constant power output exercise has also been shown to be consistent irrespective of exercise duration (between ~2 and 15 min) during both isolated muscle contractions [13, 52] and whole body exercise [15]. This consistency indicates that fatigue during both continuous and intermittent severe-intensity exercise is related to the attainment of a critical level of homeostatic disturbance, which renders exercise unsustainable or intolerable. The mean rate at which representative indices of metabolic perturbation such as [PCr], [*P*_i_], and pH change during exercise will therefore determine the tolerable duration of exercise; during intermittent exercise, the mean rate of change of these indices as well as their sequelae (e.g., adenosine and inosine monophosphate [AMP], [IMP], [Ca^2+^], and [K^+^] will naturally be influenced by *P*_W_, *D*_W_, *P*_R_, and *D*_R_. It is important to note that several substrates and metabolites that have been linked with the process of muscle fatigue are also known to stimulate mitochondrial respiration [14, 53]. Therefore, rates of change of these substrates and metabolites will be causally and temporally related to the rate of change of $\dot{V}{\text{O}}_{2}$ during severe-intensity exercise, with, for example, the minimum value of [PCr] and the maximum value of [*P*_i_], coinciding with the attainment of $\dot{V}{\text{O}}_{2\hbox{max} }$ close to the limit of tolerance. Lower recovery power outputs and/or longer recovery periods during intermittent exercise would simultaneously blunt the changes in intramuscular substrates/metabolites and the development of the $\dot{V}{\text{O}}_{2}$ slow component, therefore extending time to the limit of tolerance.

An interesting feature of the study by Chidnok et al. [51] was that [PCr] recovery kinetics were slower at the end than at the beginning of the intermittent exercise protocols. While the interpretation of these ^31^P-MRS data is made more complicated by changes in pH during exercise, this slowing of [PCr] kinetics might indicate a reduction in muscle oxidative capacity [54], which might in turn imply a reduction in CP, as fatigue ensues. Practically, slower [PCr] kinetics during the later compared with the earlier stages of an intermittent exercise protocol (or interval training session) has implications for training prescription and for modeling *W*′ reconstitution during intermittent exercise (which was not considered in earlier models [47, 48]). Similarly, possible changes in CP over time during an exercise session presents a challenge for the development of models that track *W*′ utilization and predict exercise performance.

## Refining the Model

Early studies in which the CP concept was applied to intermittent exercise [47, 48] necessarily made some assumptions and simplifications. One such assumption was that *W*′ reconstitution in recovery is a linear process with a fixed rate (in J·s^−1^) for the duration of each recovery period. However, not only is it possible that *W*′ reconstitution during recovery intervals becomes slower towards the end of a period of exhaustive intermittent exercise [51], it is also possible that *W*′ reconstitution within a recovery interval is not linear.

Ferguson et al. [55] examined the time course of *W*′ reconstitution by asking subjects to complete a series of conventional constant power output exercise bouts at discrete time points (2, 6, and 15 min) during the recovery from an initial bout of exhaustive severe-intensity exercise. In each case, the hyperbolicity of the power–time relationship was preserved, and CP did not significantly differ from that measured in the control (unfatigued) condition. In contrast, *W*′ differed significantly in each condition: ~22 kJ in the control condition and 8, 14, and 19 kJ when measured after 2, 6, and 15 min of recovery, respectively. These results are consistent with other studies that show that *W*′ but not CP is altered by prior high-intensity exercise, depending on the duration of subsequent recovery [56] and the extent of the initial *W*′ utilization [42]. From the three time points studied (2, 6, and 15 min), Ferguson et al. [55] observed that *W*′ was reconstituted more rapidly in the early than in the late recovery period, i.e., the pattern of *W*′ reconstitution appeared to be curvilinear rather than linear. These authors also noted that the time course of *W*′ reconstitution (half-time of ~234 s) was faster than that of blood lactate clearance (half time of ~1366 s) but slower than the recovery of $\dot{V}{\text{O}}_{2}$ (which they considered as a proxy for the recovery of muscle [PCr], half-time of ~74 s).

Building on the work of Ferguson et al. [55], and using the data of Chidnok et al. [48], Skiba et al. [57] applied a continuous equation to model *W*′ reconstitution kinetics during intermittent exercise, with the assumption that *W*′ equaled zero at the limit of tolerance:

2 $$ {W_{\text{BAL}}^{\prime }} = W^{\prime } -  \int\limits_{0}^{t} {W_{\exp }^{\prime }} \cdot {\text{e}}^{\frac{{-({t-u})}}{\tau_{W^{'}}}} \cdot {\text{d}}u $$

where $W_{\text{BAL}}^{\prime }$ represents the balance of *W*′ remaining, *W′* equals the known *W*′ for continuous exercise, $W_{ \exp }^{\prime }$ is equal to the expended *W*′, and (*t* *−* *u*) is equal to the time(s) between segments of the exercise session that resulted in a depletion of *W*′. The relationship was best fit with an exponential with the time constant for *W*′ reconstitution being negatively related to the difference between CP and the recovery power output, i.e., *W*′ was reconstituted more rapidly when *P*_R_ was smaller:

3 $$ \tau_{{W^{\prime } }} = 546\cdot {\text{e}}^{{( - 0.01\cdot {\text{DCP}})}} + 316 $$

where $\tau_{{W^{\prime } }}$ is the time constant for *W*′ reconstitution and DCP is the difference between CP and *P*_R_.

The time constant for *W*′ reconstitution was ~377 s when recovery occurred at 20 W, which is consistent with Ferguson et al. [55], ~452 s when recovery occurred in the moderate-intensity domain, and ~580 s when recovery occurred in the heavy-intensity domain [57]. The *W*′ reconstitution time constant increased to non-physiological values (~7056 s) when the *P*_R_ remained above CP, indicating no net recharge of *W*′ but merely a slower rate of *W*′ utilization in the recovery intervals compared with the work intervals. Moreover, the amount of *W*′ remaining at any time during intermittent exercise (i.e., *W*′ ‘balance’, $W_{\text{BAL}}^{\prime }$) was negatively related to the rise in $\dot{V}{\text{O}}_{2}$, suggesting a link between *W*′ and the $\dot{V}{\text{O}}_{2}$ slow component. As a ‘proof of principle’, Skiba et al. [57] applied the $W_{\text{BAL}}^{\prime }$ model to the power output data of a competitive cyclist during a road race. Using the cyclist’s known CP and *W*′ values, and the group mean values for the time constants of *W*′ reconstitution for power outputs below CP, Skiba et al. [57] described the time course of the dynamic utilization and reconstitution of *W*′ throughout the race, during which power output naturally fluctuated. Importantly, the near-complete utilization of *W*′ by the cyclist as predicted by the $W_{\text{BAL}}^{\prime }$ model coincided with the cyclist’s termination of exercise (retirement from the race).

**Fig. 6:**
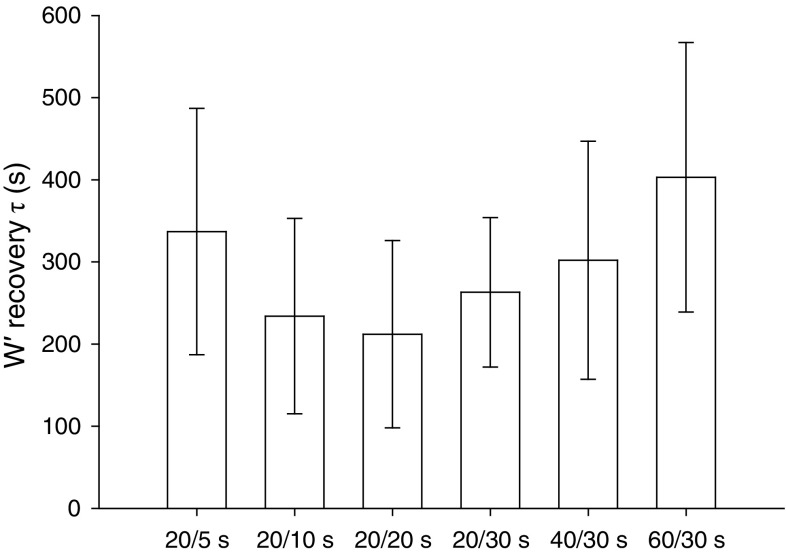
The influence of different work and recovery intervals during intermittent severe-intensity exercise on the time constant (*τ*) for *W*′ reconstitution. The mean ± standard deviation *W*′ recovery time constant tended to become shorter as the recovery duration separating 20-s work bouts was increased from 5 to 20 s. Conversely, the *W*′ recovery became progressively slower as the recovery duration was kept constant at 30 s, whereas the work duration was increased from 20 to 60 s. Figure re-drawn based on data from Skiba et al. [58]

As first described by Morton and Billat [47], the utilization and reconstitution of *W*′ will depend on both the power outputs (relative to CP) and the durations of the work and recovery intervals during intermittent exercise. Having described the relationship between recovery power output and the kinetics of *W*′ reconstitution [57], it was necessary to evaluate the effects of work and recovery interval duration on *W*′ kinetics. To challenge the $W_{\text{BAL}}^{\prime }$ model, Skiba et al. [58] asked subjects to complete severe-intensity intermittent exercise, using six different combinations of work and recovery interval durations, until they had utilized 50% of their predicted $W_{\text{BAL}}^{\prime }$. The work–rest interval durations were 20–5 s, 20–10 s, 20–20 s, 20–30 s, 40–30 s, and 60–30 s, with fixed work and recovery interval power outputs. Following each of the intermittent exercise protocols, subjects exercised at a constant severe-intensity power output until the limit of tolerance. The actual *W*′ ($W_{\text{ACT}}^{\prime }$) measured during the constant power output test was then compared with the amount of *W*′ predicted to be available by the $W_{\text{BAL}}^{\prime }$ model. The time constant of *W*′ reconstitution tended to be shorter (i.e., reconstitution was more rapid) both when work interval duration was reduced and when recovery interval duration was increased (Fig. 6), resulting in an under-prediction of $W_{\text{ACT}}^{\prime }$ and severe-intensity exercise tolerance. The time constant for *W*′ reconstitution was similar to that reported previously by Skiba et al. [57] when work interval duration was long (60–30 s, ~403 s) and when recovery interval duration was short (20–5 s, ~337 s), but was shorter than expected at the other work–recovery permutations (e.g. ~212 s at 20–20 s). Skiba et al. [58] speculated that this might be a consequence of greater muscle oxygenation during intermittent exercise, particularly in type II fibers, which might increase CP, increase *W*′, and/or speed PCr recovery kinetics during recovery intervals. However, it should be noted that, although some differences between $W_{\text{ACT}}^{\prime }$ and $W_{\text{BAL}}^{\prime }$ were found to exist, these were generally small, amounting to only ~1.6 kJ when averaged across conditions (i.e., within ~10% of *W*′). Consistent with Skiba et al. [57], $W_{\text{ACT}}^{\prime }$ was correlated with the change in $\dot{V}{\text{O}}_{2}$ between the start and the end of the constant power output exercise bout (*r* = 0.79). That $W_{\text{ACT}}^{\prime }$ was, for the most part, accurately predicted in this study indicates that variations in work and recovery durations during intermittent exercise did not adversely influence model outcomes, therefore supporting the validity of the $W_{\text{BAL}}^{\prime }$ model. The relationship between *W*′ and $\dot{V}{\text{O}}_{2}$ shown by Skiba et al. [57, 58] suggests there may be a physiologically optimal formulation of work and recovery intervals that minimizes $\dot{V}{\text{O}}_{2}$ and enhances exercise tolerance. Within the range of work and recovery interval durations studied by Skiba et al. [58], and when work and recovery intensities are fixed, it would appear that relatively short exercise durations (20 s) in conjunction with relatively short recovery durations (10–30 s) result in a low O_2_ cost of intermittent exercise and fast *W*′ reconstitution kinetics, which should theoretically extend exercise tolerance. This finding, which is consistent with evidence that contraction duty cycle influences CP [59], may have practical implications in exercise and training prescription.

To extend understanding of the physiological foundations of *W*′ and the $W_{\text{BAL}}^{\prime }$ model, Skiba et al. [60] used ^31^P- and ^1^H-MRS techniques to compare the recovery of *W*′ versus the recovery of selected intramuscular substrates and metabolites. Following determination of CP and *W*′, subjects completed severe-intensity, single leg knee-extension exercise to the limit of tolerance. They then rested in place for 1, 2, 5, or 7 min before repeating the same severe-intensity exercise bout to the limit of tolerance. With this design, the difference in the work done above CP (i.e., *W*′) between the first and second exercise bouts would indicate the extent to which *W*′ had been reconstituted in the intervening recovery interval. Skiba et al. [60] estimated the time course of *W*′ recovery and then compared it with the recovery of [PCr], pH, carnosine content, and to the output of a novel derivation of the $W_{\text{BAL}}^{\prime }$ model in which the time constant of *W*′ reconstitution was calculated as the initial *W*′ divided by the difference between CP and *P*_R_. The results indicated that muscle [PCr] recovered faster than *W*′, with time constants of ~57 and ~334 s, respectively. However, *W*′ in the second exercise bout was closely correlated with the reduction of [PCr] from the beginning until the termination of exercise (*r* = 0.99). Given the close relationship between [PCr] and $\dot{V}{\text{O}}_{2}$, these results are in accordance with earlier investigations [55, 57, 58] and indicate that the dynamics of [PCr] (and $\dot{V}{\text{O}}_{2}$) during intermittent exercise may influence *W*′ and exercise tolerance. A novel observation in Skiba et al. [60] was the inverse curvilinear relationship between muscle carnosine (a dipeptide found in high concentration in type II muscle fibers) and *W*′ reconstitution. Carnosine has mainly been considered in exercise physiology for its role in buffering pH although it may also be involved in potentiating muscle force through its interaction with calcium [61]. The inverse relationship between muscle carnosine content and the time constant of *W*′ reconstitution identified by Skiba et al. [60] warrants further investigation. Importantly, the novel $W_{\text{BAL}}^{\prime }$ model closely predicted the actual *W*′ recovery (*r* = 0.97). It was also of interest that the kinetics of *W*′ recovery in single-leg-extensor exercise [60] were generally similar to those measured in cycle exercise [58], suggesting the model may be applicable in both small and large muscle mass exercise.

Skiba et al. [62] extended their observations on a single cyclist in their original paper [57] by investigating the validity of the $W_{\text{BAL}}^{\prime }$ model in the field. Data were collected from the bicycle power meters of eight trained triathletes. For each dataset, $W_{\text{BAL}}^{\prime }$ was calculated and then compared between situations where the athletes reportedly became prematurely exhausted during training or competition and situations where the athletes successfully completed a difficult assigned task or race. Calculated $W_{\text{BAL}}^{\prime }$ differed significantly between the two situations: in the first situation, the mean $W_{\text{BAL}}^{\prime }$ at exhaustion was just 0.5 ± 1.3 kJ, which was within the standard error for measuring *W*′, whereas the minimum $W_{\text{BAL}}^{\prime }$ in the non-exhausted situation was 3.6 ± 2.0 kJ. Receiver operator characteristic curve analysis indicated that the $W_{\text{BAL}}^{\prime }$ model is useful for identifying the point at which athletes are in danger of becoming exhausted.

## Future Directions and Conclusions

Although further refinement may be required, the $W_{\text{BAL}}^{\prime }$ model developed by Skiba and colleagues [57, 58, 60] which built on earlier contributions by Morton and Billat [47], Chidnok et al. [48, 51] and Ferguson et al. [55] appears to represent an important new development in assessing athlete fatigue state and residual performance capacity during training and racing. The apparent ability of the model to track the dynamic state of *W*′ during intermittent exercise may have important implications for the planning and real-time monitoring of athletic performance. For example, a wristwatch- or handlebar-mounted monitor programmed to provide an endurance athlete with real-time feedback on the percentage of *W*′ remaining during competition could provide critical information on optimal pacing strategy (i.e., whether or not to initiate a break or to respond to an attempted break by a competitor). In future, it might also be possible for coaches to remotely monitor (using global positioning systems to provide information on speeds sustained relative to CS) the *W*′ remaining in players engaged in team sports and to use the data to inform decisions on rotations or substitutions. For these scenarios to be both possible and sufficiently precise, it would be necessary for an algorithm to be furnished with not only an individual athlete’s CP and *W*′ but also their personal time constants for *W*′ reconstitution, since this can be quite variable between individuals [57, 60]. Field-based training and testing data could potentially be used to provide this information. Another application of the $W_{\text{BAL}}^{\prime }$ model is in the development of individualized interval training sessions. With knowledge of an athlete’s CP, *W*′, and *W*′ recovery kinetics, a coach may more precisely prescribe work and recovery interval intensities and durations to achieve specific physiological goals. However, one factor not yet fully considered is the extent to which some features of the model, such as CP and the time constant for *W*′ reconstitution, might themselves be modified over time as fatigue ensues. Such changes would need to be incorporated into future formulations of the $W_{\text{BAL}}^{\prime }$ model.

In conclusion, the hyperbolic power–time relationship provides an essential foundation for understanding the physiological bases of fatigue development in different exercise intensity domains. For continuous exercise, the CP model has found many important uses in performance modeling and training prescription [9, 10, 26]. Recently, increasing attention has focused on applying the CP model to intermittent exercise. Performance during such exercise depends essentially on the individual’s CP and *W*′, the work interval power output and duration, and the recovery interval power output and duration [5]. However, whereas *W*′ may be utilized linearly when power output exceeds CP, *W*′ may not necessarily be reconstituted linearly, a factor that is explicitly accounted for in the $W_{\text{BAL}}^{\prime }$ model. Many popular team sports (e.g., basketball, football, hockey, rugby) are characterized by frequent bursts of severe-intensity exercise interspersed by lower-intensity recovery periods. The potential for application of the CP model to better understand the limitations to performance and to inform training practices in such sports is therefore quite considerable.
